# Impact of Rheumatoid Arthritis and Traditional Risk Factors on Outcomes in Acute Coronary Syndrome

**DOI:** 10.7759/cureus.86560

**Published:** 2025-06-22

**Authors:** Masi Javeed, Camila Jaramillo, Sai Santosh Sreenivasan, Rias Ali, Monicka Felix

**Affiliations:** 1 Cardiology, HCA Florida Trinity Hospital/USF Morsani College of Medicine GME, Trinity, USA; 2 Internal Medicine, HCA Florida Trinity Hospital/USF Morsani College of Medicine GME, Trinity, USA

**Keywords:** acs, autoimmune disease, cardiology, cardiovascular disease, cardiovascular disease risk, cardiovascular mortality, rheumatoid arthritis, rheumatology, rheumatology and autoimmune diseases

## Abstract

Objective

The purpose of this study was to better understand the impact of a preexisting diagnosis of rheumatoid arthritis (RA) on patient hospital outcomes in acute coronary syndrome (ACS) in comparison to traditional ACS risk factors.

Methods

This retrospective study protocol included 673 patients hospitalized with ACS in the HCA Healthcare West Florida Division from January 1, 2016, to December 31, 2023. Analysis via logistic regression and negative binomial regression compared associations between patients with ACS as primary diagnostic codes during their hospital admissions who also had RA, considering demographics like age, sex, and race. Patient encounters and diagnoses were identified using ICD-10 codes. Regression models were used for our analysis due to the straightforward computation, increased reproducibility, ability to use both categorical and continuous variables, and capability to convert diagnostic codes into binary variables. Traditional risk factors for ACS were also included in multivariate analyses. These included current tobacco use, former tobacco use, alcohol use disorder, elevated BMI, hyperlipidemia (HLD), and diabetes mellitus (DM). Pregnant patients, patients below 18 years of age, patients missing demographic information, and patients with other autoimmune conditions were excluded from the study.

Results

For RA, the odds of in-hospital mortality were not significantly 0.779 times as likely (p-value 0.2252, 95% CI (0.520, 1.167)), and 30-day readmission odds were not significantly 0.948 times as likely (p-value 0.5671, 95% CI (0.789, 1.139)). RA resulted in a 1.034-factor statistically insignificant increase in length of stay (LOS) (p-value 0.3369, 95% CI (0.965, 1.108)). For the traditional risk factors, odds of in-hospital mortality were 1.071 times as likely for every one-year increase in age (p-value <0.0001, 95% CI (1.065, 1.077)), 1.285 times as likely for current smokers (p-value 0.0020, 95% CI (1.096, 1.507)), 0.970 times as likely for every one-point increase in BMI (p-value <0.0001, 95% CI (0.961, 0.980)), 0.647 times as likely for patients with HLD (p-value <0.0001, 95% CI (0.576, 0.726)), and 1.349 times as likely for patients with DM (p-value <0.0001, 95% CI (1.212, 1.502)). Age, DM, and alcohol use disorder resulted in statistically significant increased 30-day readmission. Age, male sex, Black race, other non-Caucasian races, former tobacco use, current tobacco use, DM, and alcohol use disorder resulted in statistically significant increased LOS.

Conclusions

RA was surprisingly associated with decreased in-hospital mortality and 30-day readmission in the setting of ACS despite an associated increased LOS, which needs to be investigated further. In terms of statistical significance, there was no difference in these outcomes in patients with RA versus patients without RA. HLD was unexpectedly associated with a significant decrease in in-hospital mortality, which requires further investigation. Meanwhile, the traditional risk factors, except BMI and HLD, continued to show worse outcomes with statistical significance in the same patient population. Longitudinal follow-up and further clinical investigation of these patient encounters will likely shed more light on these associations. This knowledge may prevent over-utilization of time, equipment, and resources when addressing hospitalized patients with RA presenting with ACS, particularly in acute care settings.

## Introduction

Acute coronary syndrome (ACS) is an umbrella term that refers to a group of conditions when blood perfusion to the myocardium is suddenly reduced or blocked [1]. This includes non-ST-elevation myocardial infarction (NSTEMI), which refers to a reduction in myocardial perfusion without recognizable ST-elevation on electrocardiogram (EKG); ST-elevation myocardial infarction (STEMI), which is a reduction in myocardial perfusion with recognizable ST-elevation on EKG; and unstable angina, myocardial ischemia along with chest pain [1].

Symptoms of ACS include the most common patient complaints in the emergency department [1] and refer to an entire spectrum of etiologies [1]. As such, ACS forms one of the most common diagnoses in modern medicine. Indeed, a substantial effort is made across medicine to diagnose, treat, and screen for ACS efficiently. In both primary care and cardiac disciplines, a widespread effort is made to both further understand and discover patient risk factors that predispose patients to developing ACS. These include older age, diabetes mellitus (DM), hypertension, hyperlipidemia (HLD), smoking, and obesity [2]. ACS forms one facet of coronary artery disease, which in turn is one of the many manifestations of cardiovascular disease, which forms the bedrock of both the diagnosis and treatment of disease in cardiac disciplines [2].

ACS is a condition that poses life-threatening consequences. There is a person who suffers from ACS every 41 seconds in the United States [3]. ACS is one of the most common causes of death and is responsible for one-third of total deaths in those aged 35 and older in the United States [2]. It also affects humans of all ages and leads to an enormous economic burden on the healthcare system, as high as $150 billion annually [4].

A variety of underlying conditions pose further risk factors for developing ACS. Of these, several include autoimmune conditions such as psoriasis, rheumatoid arthritis (RA), and systemic lupus erythematosus (SLE). RA is the most common form of autoimmune arthritis. RA poses as a novel risk factor for ACS, which has not been studied extensively. Studies conducted in Norway and Sweden note a positive correlation between RA and the risk of developing ACS. The postulated theory stems from the increased predisposition of autoimmune disease patients to experience a hypercoagulable state [5]. Hypercoagulability generally increases both the viscosity of blood and the risk of developing coronary artery thrombosis and arterial occlusion, which form the basis of ACS.

Here, we have investigated whether a preexisting diagnosis of RA is associated with worsened hospital outcomes such as length of stay (LOS), 30-day readmission rates, and in-hospital mortality in the HCA Healthcare West Florida Division in the USA. These hospital outcomes are a readily accessible group of data that can serve as a general indicator of illness severity while in a common hospital system that follows similar treatment protocols. They can also be used to optimize the utilization of healthcare resources and reduce wastage.

## Materials and methods

This study was designed as a retrospective study and was determined to be IRB exempt. Initially, all encounters in the HCA Healthcare West Florida Division in the United States (regardless of age) of patients presenting with ACS (NSTEMI, STEMI, or UA) between 1/1/2016 and 12/31/2023 were reviewed; this was a total of 40,576 encounters found in the HCA clinical database. Of this, patient encounters excluded were any with missing or incomplete demographic data that pertained to the variables used in the study (1,041 encounters) and duplicate encounters, which were multiple encounters with the same individuals (3,991 encounters). There were 2,321 encounters that did not contain the risk factors or ICD-10 coding denoting the variables in this study (age of 18 years or above, sex, Black ethnicity/race, other non-Caucasian races, current tobacco use, former tobacco use, alcohol use disorder, BMI, HLD, DM, and/or RA). This left 33,223 unique patient encounters of patients satisfying the above-mentioned criteria on their first encounter, having a diagnosis of ACS, and being admitted to the hospital at all levels of care, including medical, telemetry, progressive care, and intensive care units. Pregnant patients, patients below 18 years of age, patients missing demographic information, and patients with autoimmune diseases including irritable bowel disease, psoriasis, psoriatic arthritis, ankylosing spondylitis, dermatomyositis, polymyositis, Sjögren syndrome, temporal arteritis, polyarteritis nodosa, Takayasu arteritis, Kawasaki disease, granulomatosis with polyangiitis, microscopic polyangiitis, scleroderma, SLE, Sjögren’s disease, and mixed/undifferentiated connective tissue disease were excluded from the study. Then, patients with an ICD-10 diagnosis of RA were selected. This resulted in a total number of 673 patients with RA examined in this study. Diagnoses and patient encounters included in groups were determined strictly with ICD-10 coding and not via manual review of clinical records (see Appendix).

In order to arrive at RA as the focus of this study, we analyzed the three most common autoimmune diagnoses associated with ACS found in the HCA West Florida Division database. Namely, these were RA, SLE, and psoriasis. RA was found to be the most common. We attempted to assess the difference in mean age and prevalence of risk factors between patients with RA with ACS using ICD-10 coding, the secondary objective of this study.

The association of risk factors, including RA, age, sex, Black ethnicity/race, other non-Caucasian races, current tobacco use, former tobacco use, alcohol use disorder, BMI, HLD, and DM, with in-hospital mortality and 30-day readmission (within HCA West Florida Division) was evaluated using logistic regression to predict the likelihood of a binary variable. Binary variables were defined as having a risk factor or not, with the binary value “1” assigned to having a risk factor and “0” assigned to not having the same risk factor. Log odds were then calculated for each comparison (30-day admission vs. risk factors and in-hospital mortality vs. risk factors).

LOS correlation with the aforementioned risk factors was completed using negative binomial regression. In this case, LOS was designated as the outcome variable. Similarly, binary numerals were assigned to each risk factor, and log counts of occurrences were calculated, after which results were reported as incident risk ratios (IRR), and odds ratios were reported in the logistic regression models. Statistical significance for all statistical analyses was defined as a p-value < 0.05. Logistic regression models were used for our analysis due to the straightforward computation, increased reproducibility, ability to use both categorical and continuous variables, and capability to convert diagnostic codes into binary variables.

## Results

To better understand the impact of RA on ACS, it is important to find an autoimmune condition that carries perhaps the most impact as an ACS risk factor. We surveyed the three most common autoimmune conditions found in the three categories of ACS (STEMI, NSTEMI, and UA) patients in the HCA Healthcare West Florida Division. These were SLE, RA, and psoriasis. SLE had a total of 169 patient encounters, and psoriasis had 197 total. RA was found to have a total of 673 patient encounters in the designated time frame, as seen in Table 1. It was seen that among all three categories of ACS, RA had the highest association, with 331 patient encounters associated with NSTEMI, 95 with STEMI, and 247 with UA.

**Table 1:** Frequency of RA in patients with a primary diagnosis of ACS ACS, acute coronary syndrome; NSTEMI, non-ST-elevation myocardial infarction; RA, rheumatoid arthritis; STEMI, ST-elevation myocardial infarction; UA, unstable angina

RA status	NSTEMI	STEMI	UA	Total
Yes	331	95	247	673
No	16,642	5,967	11,312	33,921

**Figure 1:**
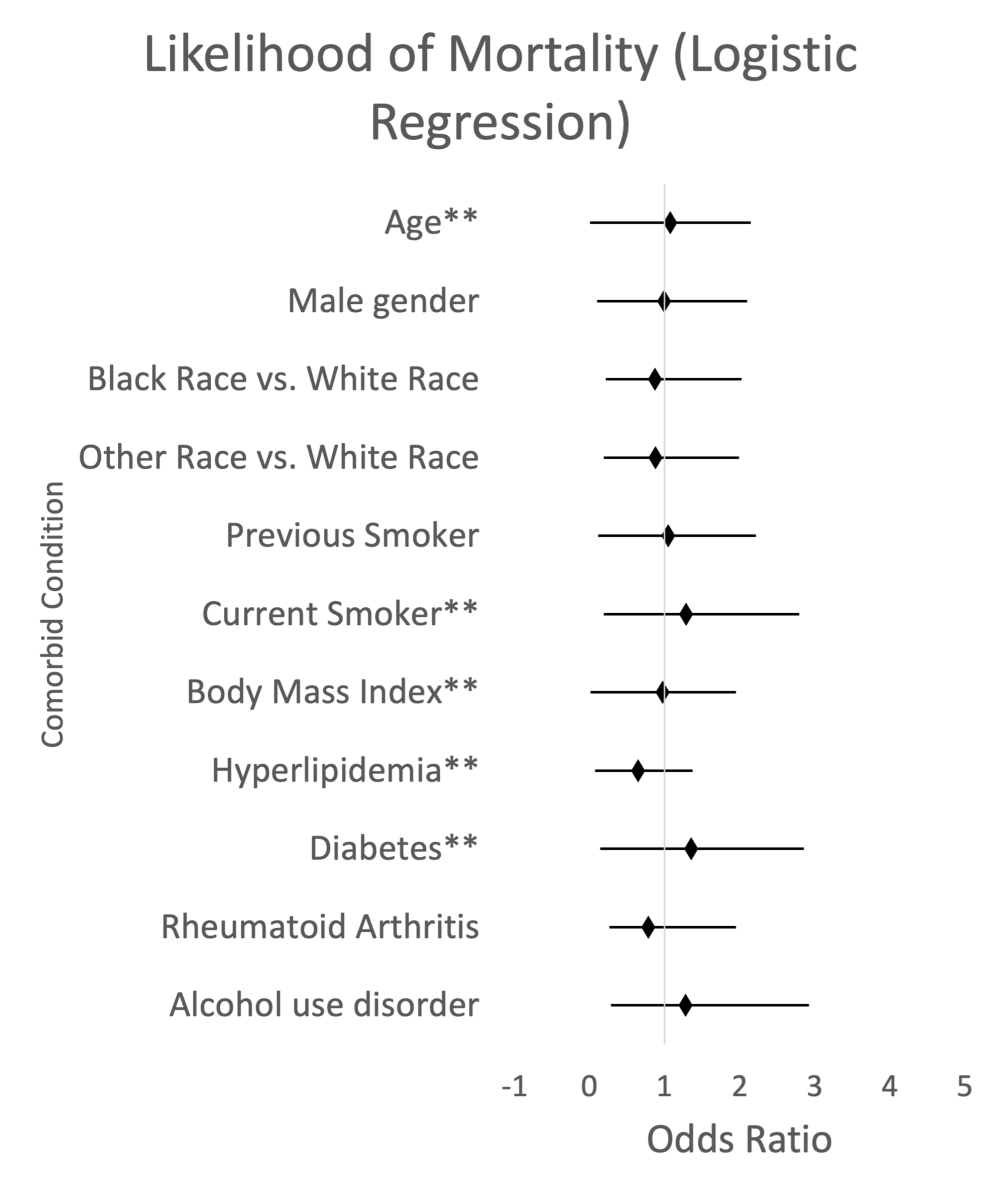
Likelihood of mortality by risk factors for ACS (95% CI) ^**^ Values with significant differences (p-value <0.05) ACS, acute coronary syndrome

When examining the odds of in-hospital mortality as displayed in Figure 1, we found that the odds of in-hospital mortality were statistically insignificant (0.779, p-value 0.2252, 95% CI (0.520, 1.167)) in the RA group when compared to statistically significant odds with every one-year increase in age (1.071, p-value <0.0001, 95% CI (1.065, 1.077)), statistically significant as well in current smokers (1.285, p-value 0.0020, 95% CI (1.096, 1.507)), and as well for every one-point increase in BMI (0.970, p-value <0.0001, 95% CI (0.961, 0.980)). Odds of in-hospital mortality association were also significant in patients with HLD (0.647, p-value <0.0001, 95% CI (0.576, 0.726)), and patients with DM (1.349, p-value <0.0001, 95% CI (1.212,1.502)).

**Figure 2:**
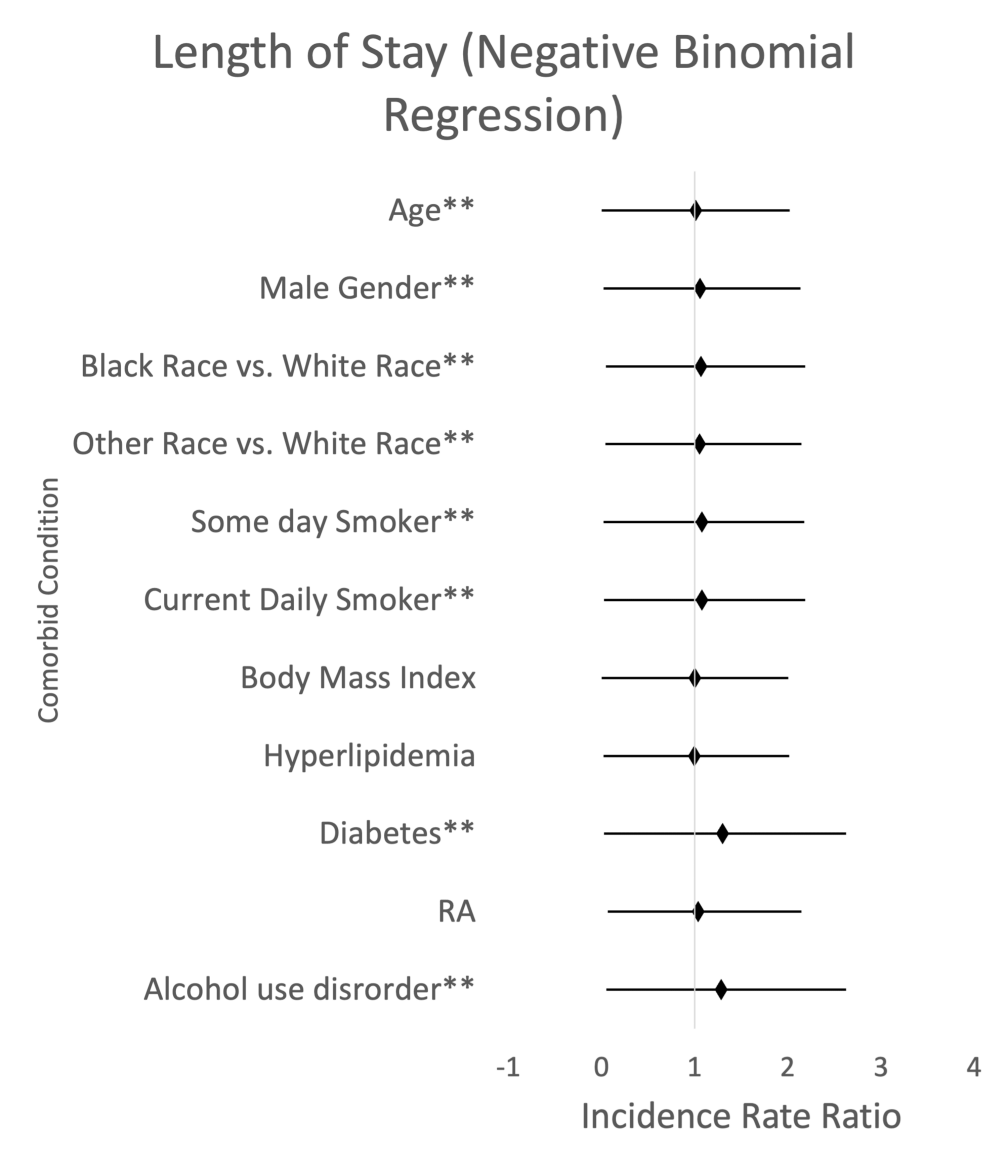
LOS by risk factors for ACS (95% CI) ^**^ Values with significant differences (p-value <0.05) ACS, acute coronary syndrome; LOS, length of stay; RA, rheumatoid arthritis

Comparison of LOS was examined with negative binomial regression, as seen in Figure 2. IRRs were obtained and are referred to here as “factors.” RA did not result in a significant factor increase in LOS (1.034, p-value 0.3369, 95% CI (0.965, 1.108)), which was not statistically significant. However, when looking at traditional risk factors, age, male sex, Black ethnicity, other non-Caucasian ethnicities, former tobacco use, current tobacco use, DM, and alcohol use disorder all resulted in statistically significant increased LOS. Age resulted in a significant increase in LOS (1.009, p-value <0.0001, 95% CI (1.008, 1.010)), as did male sex, with a 1.057-factor increase in LOS (p-value <0.0001, 95% CI (1.036, 1.078)). Black ethnicity also resulted in a statistically significant increase in LOS (1.068, p-value 0.0026, 95% CI (1.023, 1.078)), and other non-Caucasian ethnicities a significant 1.052-factor increase in LOS (p-value 0.0099, 95% CI (1.012, 1.093)). Smoking also led to a significant increase in LOS, with former tobacco use (previous smoker) a 1.078-factor increase (p-value <0.0001, 95% CI (1.050, 1.106)), and current tobacco a 1.074-factor increase (p-value <0.0001, 95% CI (1.050, 1.098)). A diagnosis of DM also led to a statistically significant increase (1.299, p-value <0.0001, 95% CI (1.273, 1.325)), and alcohol use disorder led to a statistically significant 1.283-factor increase (p-value <0.0001, 95% CI (1.229, 1.339)).

**Figure 3:**
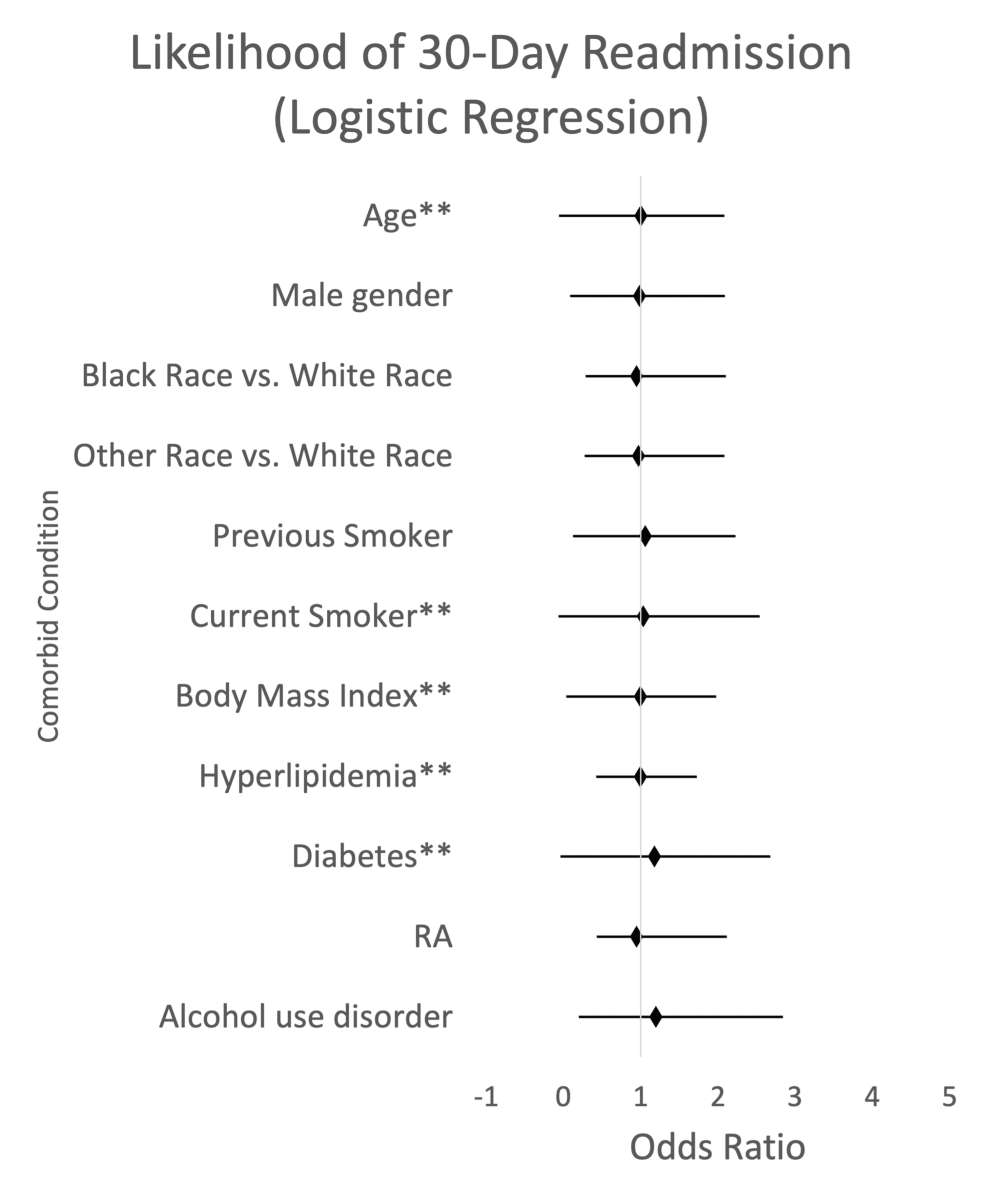
Likelihood of mortality by risk factors for ACS (95% CI) ^**^ Values with significant differences (p-value <0.05) ACS, acute coronary syndrome; RA, rheumatoid arthritis

Next, using logistic regression, we examined the odds of 30-day readmission as seen in Figure 3. RA did not result in statistically significant readmission rates (0.948, p-value 0.5671, 95% CI (0.789, 1.139)). Concerning traditional risk factors, age, DM, and alcohol use disorder resulted in statistically significant increased 30-day readmission. Regarding the increase in 30-day readmission, the following had statistically significant associations: age (for every one-year increase in age) (1.004, p-value <0.0025, 95% CI (1.001, 1.006)), DM (1.174, p-value <0.0001, 95% CI (1.114, 1.237)), and alcohol use disorder (1.197, p-value <0.0017, 95% CI (1.070, 1.338)).

## Discussion

ACS forms one of the deadliest and most common complaints among patients presenting at a hospital. Autoimmune conditions such as RA pose unique risk factors in the risk for and management of ACS. To better understand the impact of RA on ACS, it is important to find an autoimmune condition that carries perhaps the most impact as an ACS risk factor. We surveyed the three most common autoimmune conditions found in the three categories of ACS patients in the HCA Healthcare West Florida Division and found RA to be the most common, as seen in Table 1. It was seen that among all three categories of ACS, RA had the highest association, with 331 patient charts associated with NSTEMI, 95 with STEMI, and 247 with UA. Scientific literature also supports this data [5,6]. Long-term low-grade systemic inflammation increases the risk for ACS [5]. Therefore, it is postulated that repeated low-grade systemic inflammation leads to an increased risk of the cardiovascular system developing ACS. Large systematic analyses also seem to suggest that RA perhaps leads to an increased mortality of likely cardiovascular origin, at times up to 54% more [6]. As such, RA poses a novel, possibly high-impact risk factor in the development of ACS and thus was chosen as the primary focus of this study.

This retrospective study sought to investigate the impact of RA on the development of ACS when compared to traditional risk factors for ACS. When examining the odds of in-hospital mortality as displayed in Figure 1, we found that the odds of in-hospital mortality were 0.779 times as likely (p-value 0.2252, 95% CI (0.520, 1.167)) in the RA group. However, the odds of mortality were statistically significant compared to traditional risk factors. Odds of mortality with traditional risk factors were 1.071 times as likely for every one-year increase in age (p-value <0.0001, 95% CI (1.065, 1.077)), 1.285 times as likely for current smokers (p-value 0.0020, 95% CI (1.096, 1.507)), 0.970 times as likely for every one-point increase in BMI (p-value <0.0001, 95% CI (0.961, 0.980)), 0.647 times as likely for patients with HLD (p-value <0.0001, 95% CI (0.576, 0.726)), and 1.349 times as likely for patients with DM (p-value <0.0001, 95% CI (1.212,1.502)). Comparison of LOS was examined with negative binomial regression, as seen in Figure 2. RA resulted in an increase in LOS (p-value 0.3369, 95% CI (0.965, 1.108)), which was not statistically significant. However, when looking at traditional risk factors, age, male sex, Black ethnicity, other non-Caucasian ethnicities, former tobacco use, current tobacco use, DM, and alcohol use disorder all resulted in statistically significant increased LOS. When we examined the odds of 30-day readmission as seen in Figure 3, we found that for patients with RA, the odds of 30-day readmission were not statistically significant. Traditional risk factors of age, DM, and alcohol use disorder resulted in a statistically significant increase in 30-day readmission.

Systematic meta-analyses examining the correlation between RA and ACS over the last 50 years show a decreased cardiovascular mortality in patients with RA [7]. This apparent reduction in cardiovascular mortality appears to lie in the newer advances in the detection and treatment of RA [8]. In a study of a population in Minnesota, USA, a group of patients with a similar assortment of traditional risk factors for cardiovascular disease and RA were followed [8]. It was observed that patients with incidental (newly diagnosed) RA in the early 2000s had markedly lower cardiovascular mortality when measured against non-RA patients when compared to the same in RA patients diagnosed in the 1990s [8]. Ongoing research and development into the detection and treatment of autoimmune conditions may become crucial to ameliorating the risks of ACS and cardiovascular mortality in patients with autoimmune conditions. In addition, substantial research remains to be done to delineate what concomitant conditions, along with autoimmune diseases, exacerbate mortality risk.

The data from our study were somewhat contrary to the aforementioned studies [5,6], which mention an increased risk of ACS and mortality in patients with autoimmune conditions such as RA. It is a possibility that a larger sample size would lead to different results. This study was limited to the HCA Healthcare West Florida Division, which consists of a total of 15 hospitals. Some answers, however, may also lie in modern advances in the early detection and treatment of RA. New research into the role of proinflammatory cytokines such as IL-6 and TNF-α, as well as further investigations into treatments targeting TNF-α signal transduction, shows promise in both the early detection of RA and reliable therapeutic response determination of new therapies in the treatment of RA [9]. These advances will likely have an impact on the true risk RA has on patients in the development of ACS.

Our research also raises questions about prevention techniques that clinicians can employ to improve ACS prevention in those patients with an existing diagnosis of RA. This arises from external research as well. One such method, which is widely accessible and relatively affordable, is statin therapy. A literature review performed using the PubMed database has shown that statin therapy in RA patients reduces the development of ACS by primary prevention [10]. Statin therapy is an established method of primary prevention of CVD in patients with increased risk [11]. Further research into the long-term efficacy of statin therapy on patients with RA will likely give additional insight into the multitude of physiological impacts of RA on patients at risk of developing CVD. External research leads us to one such area of possible research: into lipoprotein alleles that affect lipid metabolism at a genetic level [12].

Limitations

It is not surprising that traditional ACS risk factors such as DM, older age, and current tobacco use were associated with poor outcomes. These risk factors are well studied and heavily influence the surveillance and treatments for cardiovascular disease in patients at risk for ACS or post-ACS, including dual antiplatelet therapy, statins, and beta blockers [13]. Unexpectedly, increased BMI and HLD were associated with better outcomes compared with patients with RA. This association suggests a need for further investigation. The role of adiposity in the development of cardiovascular disease has been established [14]. Randomization studies performed in the United Kingdom show a significant correlation between adiposity (measured by fat mass index) and the odds of developing aortic valve stenosis and other cardiovascular conditions [14]. This is attributed to the direct impact of atherosclerosis on the development of hypoperfusion of the myocardium, which is the very definition of ACS [1].

Potential confounding variables also pose a limitation on the extent of our analysis. In all patients, medication use can affect results. It is common knowledge that various pharmacological therapies have side effects, and these side effects may have physiological effects that are both patient-specific and still largely unknown. Due to the nature of our study being based on analysis using ICD-10 coding, medication use was not controlled for in this study. Severity of RA could also potentially confound results. While ICD-10 codes used in this study include seronegative RA, severity was not measured among the included patient encounters. Severity of illness affects both pathophysiology and response to medication, which can also be patient-specific. It is commonly understood that medications and disease severity can present differently and uniquely based on each individual patient. The validity of the results could potentially be bolstered by patient follow-up. Longitudinal follow-up of the patients included in this study could provide more answers regarding both the development of the associations we analyzed and an opportunity to assess the severity of RA in patients at various time points.

It is possible as well that diets and nutritional health play a factor in the potency of the risk that BMI and HLD pose in the development of cardiovascular disease. It is postulated that vegetarian diets can improve lipid profiles in patients, thereby decreasing their risk for cardiovascular disease [15]. Perhaps more research into the effect of diets or investigating trends in serum lipid values in our target population could lead to further discoveries regarding the impacts of HLD and BMI on outcomes for RA patients who develop ACS and a better understanding of the association found in our results.

## Conclusions

RA was surprisingly associated with decreased in-hospital mortality and 30-day readmission in the setting of ACS despite an associated increased LOS, which needs to be investigated further. In terms of statistical significance, there was no difference in these outcomes in patients with RA versus patients without RA. HLD was unexpectedly associated with a significant decrease in in-hospital mortality, which requires further investigation. Meanwhile, the traditional risk factors, except BMI and HLD, continued to show worse outcomes with statistical significance in the same patient population. Longitudinal follow-up and further clinical investigation of these patient encounters will likely shed more light on these associations. This knowledge may prevent over-utilization of time, equipment, and resources when addressing hospitalized patients with RA presenting with ACS, particularly in acute care settings. 

## Appendices

ICD codes and related diagnoses used in statistical analyses

Hyperlipidemia (HLD)

• ICD 10 Code E78.00: Pure hypercholesterolemia, unspecified.

• ICD 10 Code E78.1: Pure Hypertriglyceridemia.

• ICD 10 Code E78.2: Mixed hyperlipidemia.

• ICD 10 Code E78.49: Other hyperlipidemia.

• ICD 10 Code E78.5: Hyperlipidemia, unspecified.

Diabetes (E10, E11)

• excluding:

• E10.641, E10.11 (comas)

SLE (M32 EXCEPT: M32.0 drug-induced)

HIV B20

RA (M06.9, M06.00 seronegative, M05.9 seropositive)

Psoriasis L40

Ankylosing spondylitis M45.9

Dermatomyositis M33.10

Polymyositis M33. 2

Psoriatic arthritis L40.52

Sjogren syndrome M35.00

Temporal arteritis M31.6

Polyarteritis nodosa M30.0

Takayasu arteritis M31.4

Kawasaki disease M30.3

Granulomatosis with polyangiitis M31.30

Microscopic polyangiitis M31.7

Scleroderma M34.89

Unstable Angina (UA)

I20.0

Unstable angina

I25.110

Atherosclerotic heart disease of native coronary artery with unstable angina pectoris

I25.700

Atherosclerosis of CABG(s), unspecified, with unstable angina pectoris

I25.710

Atherosclerosis of autologous vein CABG(s) with unstable angina pectoris

I25.720

Atherosclerosis of autologous artery CABG(s) with unstable angina pectoris

I25.730

Atherosclerosis of nonautologous biological CABG(s) with unstable angina pectoris

I25.750

Atherosclerosis of native coronary artery of transplanted heart with unstable angina

I25.760

Atherosclerosis of bypass graft of coronary artery of transplanted heart with unstable angina

I25.790

Atherosclerosis of other CABG(s) with unstable angina pectoris

STEMI/NTEMI

I21

Acute myocardial infarction

I21.0

ST elevation (STEMI) myocardial infarction of anterior wall

I21.01

ST elevation (STEMI) myocardial infarction involving left main coronary artery

I21.02

ST elevation (STEMI) myocardial infarction involving left anterior descending coronary artery

I21.09

ST elevation (STEMI) myocardial infarction involving other coronary artery of anterior wall

I21.1

ST elevation (STEMI) myocardial infarction of inferior wall

I21.11

ST elevation (STEMI) myocardial infarction involving right coronary artery

I21.19

ST elevation (STEMI) myocardial infarction involving other coronary artery of inferior wall

I21.2

ST elevation (STEMI) myocardial infarction of other sites

I21.21

ST elevation (STEMI) myocardial infarction involving left circumflex coronary artery

I21.29

ST elevation (STEMI) myocardial infarction involving other sites

I21.3

ST elevation (STEMI) myocardial infarction of unspecified site

I21.4

Non-ST elevation (NSTEMI) myocardial infarction

I21.9

Acute myocardial infarction, unspecified

I21.A

Other type of myocardial infarction

I21.A1

Myocardial infarction type 2

I21.A9

Other myocardial infarction type
